# Aspirin enhances opsonophagocytosis and is associated to a lower risk for *Klebsiella pneumoniae* invasive syndrome

**DOI:** 10.1186/1471-2334-14-47

**Published:** 2014-01-30

**Authors:** Chen-Hsiang Lee, Lin-Hui Su, Jien-Wei Liu, Chia-Chi Chang, Rong-Fu Chen, Kuender-D Yang

**Affiliations:** 1 Division of Infectious Diseases, Kaohsiung Chang Gung Memorial Hospital, Kaohsiung, Taiwan; 2 Chang Gung University College of Medicine, Kaohsiung, Taiwan; 3 Department of Laboratory Medicine, Chang Gung Memorial Hospital, Linkou, Taiwan; 4 Chang Gung University, College of Medicine, Kweishan, Taoyuan, Taiwan; 5 Department of Medical Research, Show Chwan Memorial Hospital, 6 Lukong Road, Lugang, Changhua 505, Taiwan

**Keywords:** Salicylate, *K. pneumoniae*, Hypermucoviscosity, Phagocytosis, Invasive syndrome

## Abstract

**Background:**

*Klebsiella pneumoniae* (KP) expressing hypermucoviscosity phenotype (HV-KP) has abundant capsular polysaccharide (CPS) and is capable of causing invasive syndrome. Sodium salicylate (SAL) reduces the production of CPS. The study was aimed to investigate the relationship between aspirin usage and KP-mediated invasive syndrome and the effect of SAL on HV-KP.

**Methods:**

Patients with community-acquired KP bacteraemia were prospectively enrolled. KP-M1, a serotype-K1 HV-KP clinical isolate, was used in the following experiments: CPS production, HV-KP phenotype, and the effect of SAL on neutrophils phagocytosis. The effect of oral aspirin intake on the leukocyte bactericidal activity was evaluated.

**Results:**

Patients infected by HV-KP and diabetic patients with poor glycemic control were at an increased risk for invasive syndrome (*p* < 0.01); those who had recent use of aspirin (*p* = 0.02) were at a lower risk. CPS production was significantly reduced in the presence of SAL. The HV-KP phenotype and resistance to neutrophil phagocytosis were both significantly reduced in the KP-M1 after incubation with SAL (*p* < 0.01). Aspirin treatment significantly enhanced the killing of KP-M1 by leukocytes (*p* < 0.01).

**Conclusion:**

Treatment with SAL significantly reduces CPS production in HV-KP, thereby contributing to leukocyte phagocytosis and bactericidal activity against this pathogen.

## Background

Primary *Klebsiella pneumoniae* (KP) liver abscess occurs most often in patients with type 2 diabetic mellitus (DM), and can present with the following metastatic complications: bacteraemia, meningitis, endophthalmitis, and necrotising fasciitis [1,2]. Glistening mucoid colonies with a viscid consistency are usually formed by KP that are cultured on agar media [1-3]. Hypermucoviscous KP (HV-KP) causes a unique constellation of symptoms, and is associated with the development of invasive syndrome [2,3]. The HV-KP strain is resistant to phagocytosis by neutrophils, an important characteristic that may contribute to the dissemination of infection [4-6]. The capsular polysaccharide (CPS) contributes to the mucoid phenotype, and has been identified as a determinant of KP infection, suggesting that CPS plays a role in the development of invasive syndrome [7,8]. CPS also has been used to develop a serotyping system for KP isolates, and currently 77 capsular serotypes have been identified. Compared to those belonged to non-K_1_ serotypes, isolates of the serotype K_1_ were significantly more virulent and more likely to be associated with KP-mediated invasive syndrome [2,7].

Chemotherapeutic agents that reduce CPS production may be effective as an adjunct therapy for HV-KP infection. Therapeutically safe concentrations of sodium salicylate (SAL), the major metabolite of aspirin, can reduce CPS production by up to 70% [9,10]. Aspirin also reduces the synthesis of prostaglandins (PGs) by inhibiting cyclooxygenase (COX)-mediated arachidonic acid metabolism [11], and therefore has attracted attention as a means of facilitating the killing of bacteria by leukocytes [12,13]. We hypothesized that SAL may enhance the phagocytosis of HV-KP by leukocytes as a result of reduced CPS production*.* In the present study, we tried to examine this hypothesis by investigating the effect of SAL on bacterial survival, hypermucoviscosity, CPS production, leukocyte phagocytosis, and bactericidal activity against HV-KP. Furthermore, we conducted a prospective study to evaluate the relationship between aspirin usage and KP-mediated invasive syndrome.

## Methods

### Ethics statement

All the protocols used in the present study have been approved by the Institutional Review Board at Kaohsiung Chang Gung Memorial Hospital (KCGMH; approval nos. 97-0599B and 100-0629B). Informed consent was not required in participants because of the observational nature of the study (97-0599B). In addition, 5 healthy male volunteers provided their written informed consents to participate in the study (100-0629B).

### Study design and participants

In this prospective study, patients who were admitted to the KCGMH between January 1, 2008, and December 31, 2010, with community-acquired mono-microbial bacteraemia caused by KP were enrolled for the investigation of risk factors for KP-mediated invasive syndrome. Only the KP isolates from the participants’ first blood collection were used in this study. Peripheral blood samples were also collected from 5 healthy male volunteers aged between 25 and 40 years old and used in the leukocyte phagocytosis and bactericidal activity assays as described below.

The diagnosis of KP-mediated invasive syndrome was made when the criteria for sepsis were met [14] plus the presence of at least one of the following infections: liver abscess, meningitis, empyema, mycotic aneurysm, necrotising fasciitis or endophthalmitis [15]. To investigate the risk factors of KP-mediated invasive syndrome, the following clinical variables were assessed: age, sex, comorbidities (including DM, liver cirrhosis, malignancy, chronic renal failure, and biliary tract disease), and a history of receiving proton-pump inhibitors or aspirin or antibiotics in the previous month prior to the collection of their first KP-positive blood culture. To monitor the glycemic control [16], the hemoglobin A1c (HbA_1c_) was checked when diabetic patients were enrolled in this study. Controls were retrieved from a computer-aided selection of eligible individuals who community onset bacteremia caused by other than KP. Each subject was age-matched, and the ratio of case of KP invasive syndrome to control patients was 1:1.

### Reagents

We purchased 10 mg/mL SAL, 50 mM ethylenediamine tetra-acetic acid, and 25 mM ethylene-bis (oxyethylenenitrile) tetra-acetic acid from Sigma-Aldrich (St. Louis, MO). All stock solutions were prepared in deionized water, brought to pH 7.5 using 10 N NaOH, and filter sterilized. Trypticase soy agar (TSA) and broth (TSB) media (Becton Dickinson, Franklin Lakes, NJ) were used for bacterial cultures. In some experiments, various concentrations of SAL were added into the growth media as indicated in the following experiments. The pH of the growth media with and without SAL was not significantly different.

### Bacterial isolates, hypermucoviscosity phenotype, and serotype determination

All KP isolates were identified using standard methods. The HV phenotype was identified using a modified string test [17], with a viscous string longer than 10 mm as the criteria for a positive result. K_1_ serotyping was assessed by polymerase chain reaction and capsular swelling technique [1]. A KP-M1 (serotype K_1_) strain was isolated from an enrolled patient with invasive syndrome presenting as liver abscess and endophthalmitis and was used as the representing strain in the following experiments.

### Effects of SAL on live KP

To exclude the possibility that SAL possesses direct bactericidal properties against KP, broth cultures of KP-M1 were incubated in the presence of various concentrations (0, 10, 30 and 300 μg/mL) of SAL. After 2, 4, 8, 10, 12, and 24 hours, the number of surviving bacteria was monitored by reading the absorbance at 490 nm. We further investigated the SAL effect on the hypermucoviscosity of KP. The KP-M1 colonies were cultured in plate media containing 0, 10, or 30 μg/mL of SAL, respectively, and incubated overnight at 37°C. The length of the mucoviscous string was measured [17].

### Quantification of CPS

The CPS concentration was determined by a modified carbazole assay for uronic acid (UA) [18] after total CPS was quantitatively extracted from whole bacterial cultures [19]. A 0.5-mL sample was mixed with 3 mL of 0.025 M sodium tetraborate (VWR, Radnor, PA) in sulfuric acid, and heated at 100°C for 10 min. After cooling, 0.1 mL of 0.125% carbazole (Sigma-Aldrich, St. Louis, MO) in absolute ethanol was added, and the samples were heated for another 15 min. The absorbance of the sample at 530 nm was measured, and the concentration of UA was extrapolated from a standard curve that was constructed using glucuronolactone (Sigma-Aldrich, St. Louis, MO) standards. To quantify CPS after SAL treatment, bacterial colonies were cultured in media containing 0, 10, or 30 μg/mL SAL and incubated overnight at 37°C. All samples were assayed at least 3 times. The quantities of CPS detected were expressed as micrograms of UA per 10^11^ colony forming units (μg UA/ 10^11^ cfu).

### Flow cytometric analysis of leukocyte phagocytosis

The KP-M1 bacteria were cultured overnight at 37°C on TSA media containing 0, 10, or 30 μg/mL SAL. The bacterial colonies were irradiated with ultraviolet light then were sub-cultured in plate to confirm sterile after overnight incubation. The irradiated cultures were resuspended in carbonate buffer containing 0.1% fluorescein isothiocyanate (FITC). The FITC-stained KP (FITC-KP) cells were counted using a bacterial cytometer and a fluorescence microscope. The FITC-KP cells were analyzed using a FACS Calibur flow cytometer (BD Biosciences, San Jose, CA) to verify that bacterial staining was uniform throughout each sample.

Phagocytosis was measured using a standard assay [20]. Neutrophil suspensions and pooled human sera were collected from healthy human volunteers. The neutrophil suspension was adjusted to contain 1 × 10^6^ cells in 100 μL PBS and was then combined with 600 μL PBS and 100 μL pooled human serum (10% v/v opsonization). Multiple volumes of 200 μL of FITC-KP cells containing approximately 4 × 10^7^ cfu/mL were added to 800 μL of the neutrophil-containing PBS to produce a final volume of 1.0 mL at a multiplicity of infection of 10:1. Each tube was incubated in a shaking water bath at 37°C for 60 min. Ethidium bromide was added to suppress extracellular fluorescence. A FACScan instrument (BD Biosciences, San Jose, CA) was used to detect FITC fluorescence at 488 nm, and 2 × 10^4^ cells were processed using the Cellquest version 1.0 software (BD Biosciences, San Jose, CA). By assessing phagocytosis of unstained and FITC-stained bacteria, the boundary of positive and negative fluorescence was established, and the percentage of ingested bacteria was determined.

### *Ex vivo* human leukocyte bactericidal activity assay

Bactericidal activity was measured using a standard assay method [21]. The KP-M1 organisms were cultured on TSA media containing 0 or 30 μg/mL SAL, respectively, and incubated overnight at 37°C. The KP-M1 organisms were opsonized by the addition of 10% pooled human serum collected from 5 healthy male volunteers who did not take any aspirin prior to the donation. The suspension was mixed at a multiplicity of infection of 10:1 to whole blood leukocytes, which had been collected from volunteers either before or one hour after a 100-mg oral dose of aspirin. Samples were collected one hour later, and diluted in H_2_O (pH 11.0, adjusted by NaOH) to lyse the leukocytes and disperse the bacteria for the power-plate colony assay [22]. All tests were performed in triplicate to ensure reproducibility.

### Statistical analysis

The categorical variables were compared using the chi-square test or the Fisher exact test, as appropriate. A multivariate logistic regression model was used to evaluate risk factors for invasive syndrome by calculating the odds ratio (OR) and 95% confidence interval (CI) of each clinical variable. All experimental data were expressed as the mean ± standard deviation. Differences among the results of the string test (mm) and phagocytosis (gate %) were analyzed using the Mann-Whitney U-test. The counts of surviving bacteria were analyzed using the log rank test. All statistical analyses were two-sided, and a *p* value less than 0.05 was considered to indicate a statistically significant result.

## Results

### Risk factors for KP-mediated invasive syndrome

Of the 408 patients with community-acquired mono-KP bacteremia, 76 (18.6%) had invasive syndrome. Based on results of the modified string test, the HV phenotype was identified in 147 (36.0%) isolates. As demonstrated in Table 1, invasive syndrome occurred more often in HV-KP-infected patients (*p* < 0.01). A large proportion (75, 42.9%) of the patients studied also suffered from DM. The proportion of KP infection-related invasive syndrome was significantly higher among these DM patients (*p =* 0.04), especially those with poor glycemic control (HbA_1c_ ≥ 9%; *p <* 0.01). Aspirin therapy received during the month prior to the diagnosis of the KP infection was found in 58 (14.2%) patients, and they were less likely to develop invasive syndrome (*p <* 0.01; Table 1). Further multivariate analysis indicated that community-acquired KP-bacteremic patients who were infected by strains expressing the HV phenotype (odds ratio [OR], 31.07; 95% confidence interval [CI], 13.55-71.22; *p* < 0.01) and DM patients with poor glycemic control (OR, 2.46; 95% CI, 1.27-4.77; *p* < 0.01) were at increased risk, whereas those who had a recent use of aspirin (OR, 0.17; 95% CI, 0.04-0.79; *p =* 0.02) were at a lower risk of acquiring KP-associated invasive syndrome. Comparing with age-matched control group (Table 1), it revealed that patients with KP-associated invasive syndrome were more likely to have DM (*p =* 0.01), DM with poor glycemic control (*p =* 0.02) and less likely to have malignancy (*p <* 0.01).

**Table 1:** **Comparisons of differences between patients with community-acquired *K. pneumoniae *** **bacteremia and control group**

	**Community-acquired *K. pneumoniae *** **bacteremia**				
**Variable**	**Invasive syndrome (%)**				
	**Yes (n = 76)**	**No (n = 332)**	***p*** **value**	**Control (%) (n = 76)**	***p*** **value**^**+**^
Hypermucoviscosity phenotype of *K. pneumoniae*	69 (90.8)	78 (23.5)	< 0.01*		
Male	40 (52.6)	150 (45.2)	0.25	40 (52.6)	0.99
Age ≥ 60 yrs	39 (51.3)	128 (38.6)	0.05	39 (51.3)	0.99
Diabetes mellitus (DM)	41 (53.9)	134 (40.4)	0.04	25 (32.9)	0.01
DM with poor glycemic control (HbA_1c_ ≥ 9%)	32 (42.1)	75 (22.6)	< 0.01*	17 (22.4)	0.02
Cardiovascular diseases	17 (22.4)	65 (19.6)	0.69	21 (27.6)	0.57
Liver cirrhosis	5 (6.6)	45 (13.6)	0.12	10 (13.2)	0.28
Chronic renal failure	10 (13.2)	35 (10.5)	0.54	18 (23.7)	0.14
Malignancy	4 (5.3)	33 (9.9)	0.27	18 (23.7)	< 0.01
Biliary tract diseases	2 (2.6)	20 (6.0)	0.39	8 (10.5)	0.10
Absence of underlying diseases	12 (15.8)	44 (13.3)	0.69	9 (11.8)	0.64
Therapy in the month prior to the infection					
Proton-pump inhibitors	8 (10.5)	24 (7.2)	0.47	15 (19.7)	0.17
Aspirin	2 (2.6)	56 (16.9)	< 0.01*	8 (10.5)	0.10
Antibiotics	5 (6.6)	28 (8.4)	0.76	12 (15.8)	0.12

^**+**^Age-matched analysis (*K. pneumoniae* invasive syndrome vs. control group).

*Results of multivariate analysis indicated that community-acquired *K. pneumoniae* bacteremic patients who were infected by strains expressing the hypermucoviscosity phenotype (odds ratio [OR], 31.07; 95% confidence interval [CI], 13.55-71.22; *p* < 0.01) and diabetic patients with poor glycemic control (OR, 2.46; 95% CI, 1.27-4.77; *p* < 0.01) were at increased risk, whereas those who had recent therapy with aspirin (OR, 0.17; 95% CI, 0.04-0.79; *p =* 0.02) were at lower risk of acquiring *K. pneumoniae*-associated invasive syndrome.

### Effects of SAL on live KP

**Figure 1:**
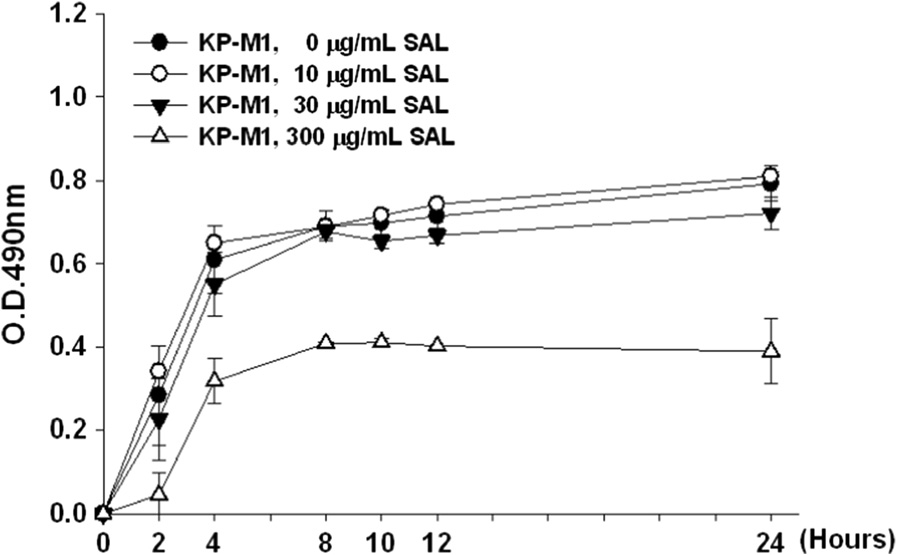
**Effects of sodium salicylate (SAL) on live KP-M1 strain of *Klebsiella pneumoniae*.** After 24-h incubation with various concentrations of SAL, the growth of KP-M1 cells were monitored by reading the absorbance (OD) at 490 nm. The growth of KP-M1 cells cultured in media containing 10 or 30 μg/mL SAL did not differ from those cultured without SAL. However, the KP-M1 cells decreased in growth when the bacteria were cultured in the presence of 300 μg/mL SAL.

No significant difference was found between the number of viable KP-M1 cells cultured with or without the presence of various concentrations of SAL. However, compared to those grown in other concentrations of SAL, viable KP-M1 cells were much less in 300 μg/mL of SAL (Figure 1). It suggests that at high concentration of SAL (300 μg/mL) might interfere with some cellular processes or bind to essential elements, which results in reduction in growth.

### Effect of SAL on hypermucoviscosity and CPS production

**Figure 2:**
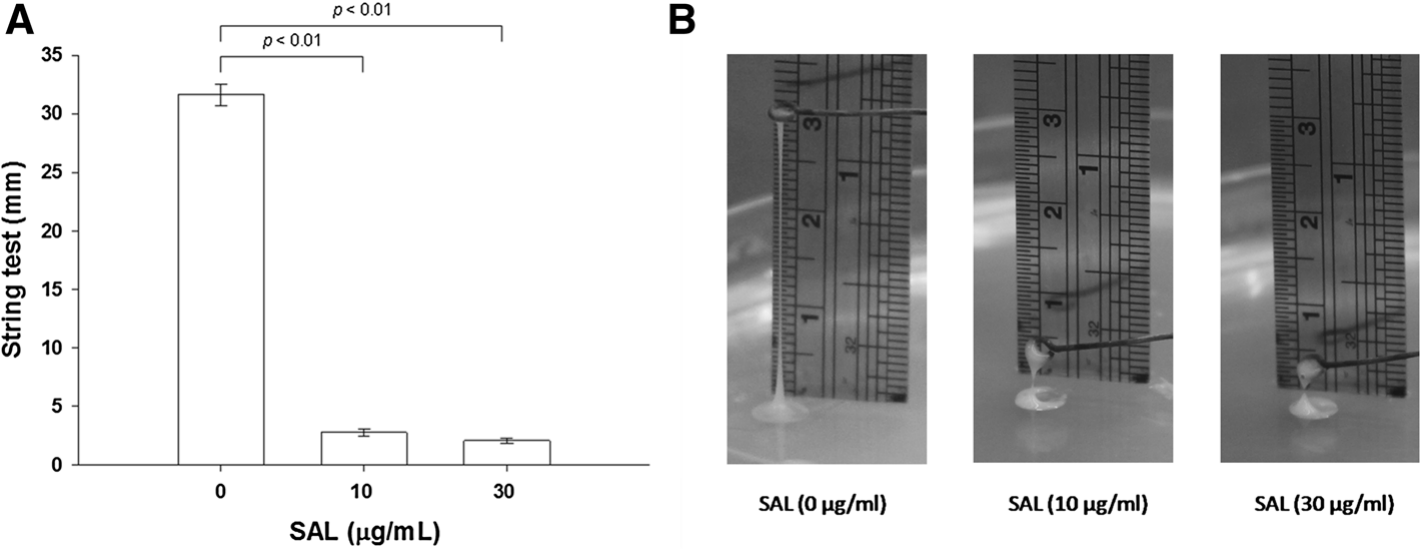
**String test for hypermucoviscosity (HV) in the KP-M1 strain of *Klebsiella pneumoniae. *(A)** Each closed bar indicates the mean ± standard deviation in millimeters for the results of string tests among KP-M1 organisms treated with various concentrations of sodium salicylate (SAL) as indicated. The asterisk indicates the significant changes among the results with different SAL concentrations (*p* < 0.01). **(B)** Representative photographs of the stretched, mucoviscous string from single colonies of KP-M1 organisms treated with various concentrations of SAL as indicated.

Incubation of KP-M1 colonies on TSB agar media containing 10 or 30 μg/mL SAL significantly decreased the incidence of the HV phenotype, according to the results of the modified string test (*p* < 0.01, Figure 2A and 2B). The quantity of CPS found in KP-M1 cells was equivalent to 2475.87 ± 175.09 μg UA/10^11^ cfu. When the KP-M1 bacteria were cultured in 10 or 30 μg/mL SAL, the quantity of CPS decreased to 1821.27 ± 73.76 μg UA/10^11^ cfu and 1574.48 ± 86.90 μg UA/10^11^ cfu, respectively. This equaled to 26.4% (*p* < 0.01) and 34.4% (*p* < 0.01) reduction of CPS levels, respectively, as compared with the mean control values. Therefore, the quantity of CPS could be significantly reduced in the presence of SAL.

### Effects of SAL on leukocyte phagocytosis and *ex vivo* leukocyte bactericidal activity

**Figure 3:**
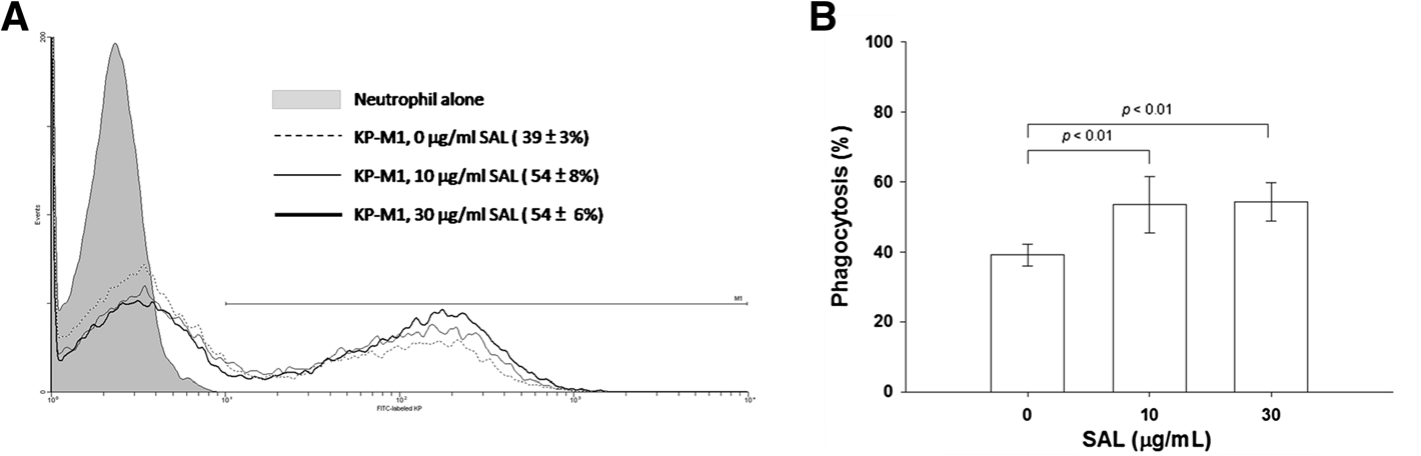
**Flow cytometry assessment of neutrophil phagocytosis. (A)** Trypan blue quenching of the extracellular fluorescence of bound bacteria and phagocytosed bacteria by neutrophils after incubation at 37°C for 60 min were evaluated by flow cytometry. **(B)** The KP-M1 bacteria were significantly more susceptible to phagocytosis by neutrophils after incubation with 10 or 30 μg/mL sodium salicylate (SAL).

**Figure 4:**
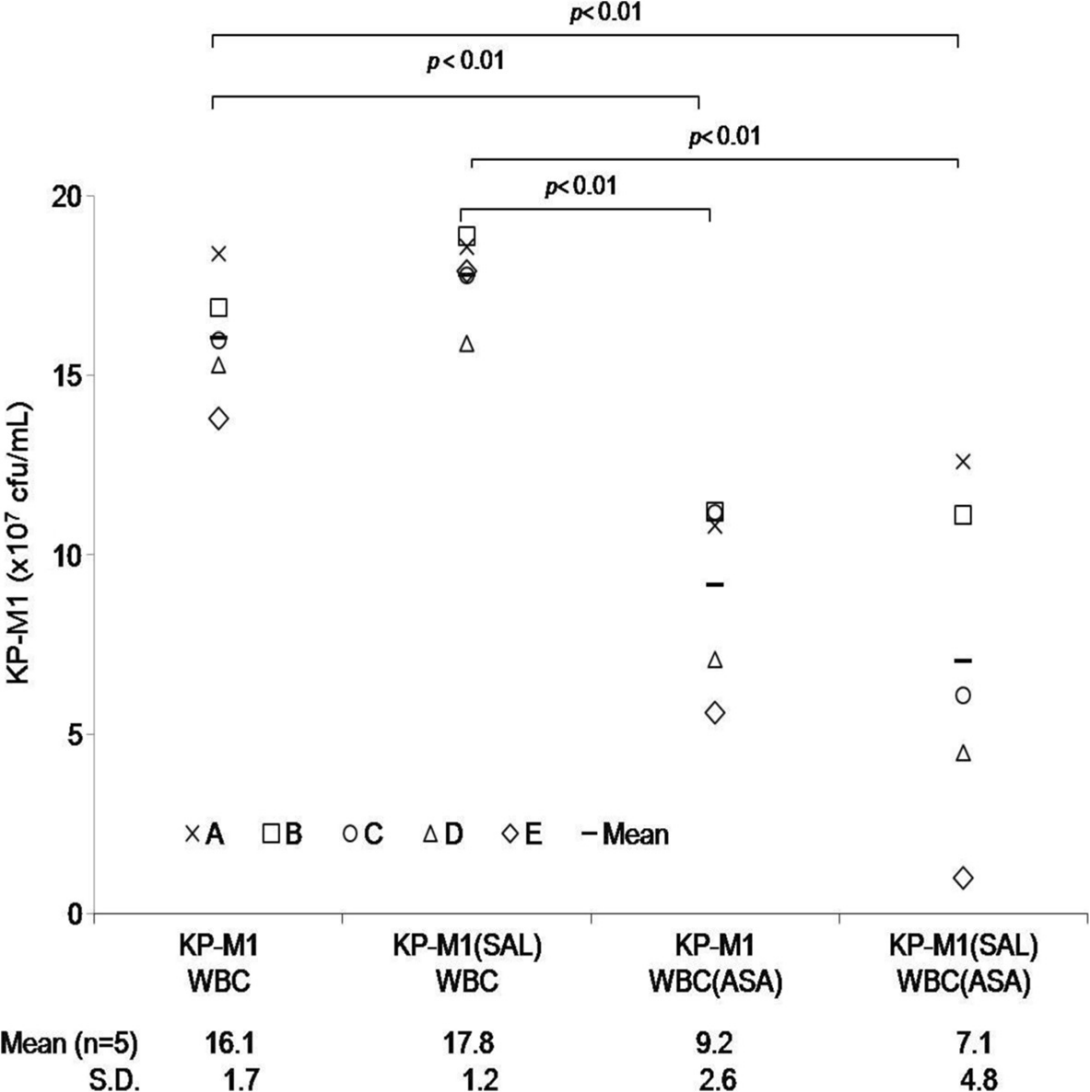
***Ex vivo *human leukocyte bactericidal activity assay.** Human peripheral white blood cells (WBCs) from 5 healthy males were collected before and one hour after oral administration of 100 mg of aspirin (ASA), and incubated, respectively, with the KP-M1 strain of *Klebsiella pneumoniae* (KP) which had been previously opsonized with normal human serum. The ASA treatment significantly enhanced the leukocyte killing of KP-M1 (*p* < 0.01), disregard whether or not the KP-M1 organisms were pre-incubated with sodium salicylate (SAL, 30 μg/mL).

The difference between the rate of KP-M1 phagocytosis by neutrophils before and after co-incubation with 10 or 30 μg/mL SAL was significant (*p* < 0.01; Figure 3A and 3B). The KP-M1 was significantly more susceptible to phagocytosis after incubation with 10 or 30 μg/mL SAL. The *ex vivo* bactericidal activity of leukocytes from healthy volunteers that were collected one hour following the oral administration of 100 mg of aspirin was significantly greater than that of leukocytes collected from the same volunteers before the aspirin treatment (*p* < 0.01; Figure 4). The results indicated that aspirin was able to enhance significantly the whole blood leukocyte bacterial killing despite the pre-incubation of KP-M1 with SAL.

## Discussion

The CPS surrounding KP bacteria enables the bacteria to evade phagocytosis [23,24]. Reduction of the CPS by SAL may therefore attenuate the pathogenicity of KP strains. Cell death could not explain the decrease in CPS observed in our experiments because viable bacterial colonies remained unchanged in the presence of various levels of SAL that were optimized for reducing the CPS. These levels of SAL are within the therapeutic range measured in serum after ingestion of low dose aspirin [25]. Hence, with the common oral use of aspirin, the effect of CPS reduction in KP could occur.

Epidemiological studies have demonstrated that the majority of KP infections are preceded by the colonization of the gastrointestinal tract [26]. Patients infected with HV-KP strains and DM patients with uncontrolled glycemia are known to be at increased risks for invasive syndrome [3,17]. Our results indicated that patients who had used aspirin during the month prior to the diagnosis of community-acquired KP bacteremia appeared to be associated with a lower risk for invasive syndrome.

The mechanism by which CPS is reduced in the presence of SAL remains unclear. Metal ions are cofactors for some of the enzymes involved in polysaccharide synthesis [27]. SAL has been identified as the biosynthetic precursor of the aromatic ring in pyochelin, an iron-chelating siderophore [28]. Divalent cations also play an important role in the stabilization of bacterial outer-membrane proteins [28]. The reduction of CPS production by SAL is likely the result of either the modulation of enzyme activity in CPS synthesis pathways or the disruption of the functional integrity of KP outer membranes.

Neutrophils have been shown to be capable of ingesting and killing encapsulated KP in the presence of complement [23]. SAL-mediated capsular suppression was shown to enable serum complement, such as C3, to bind to sites on the bacteria that were otherwise masked by the capsule [10]. In our experiments, homogenization with SAL removed 20% to 40% of the CPS production and resulted in approximately 40% to 60% increase in the rate of phagocytosis. Although homogenization with low doses of aspirin did not completely remove the capsule, it may render the bacteria to become more susceptible to phagocytosis by neutrophils. The loss of capsular materials may help to expose the cell surface of KP to the host defense mechanisms and thus shorten the time required for the clearance of the infection [29].

Recently, aspirin was shown to promote the killing of *S. pneumoniae* by blood leukocytes through COX inhibition [13]. Similarly, in the present study, we further demonstrated that aspirin enhanced the killing of HV-KP by leukocytes. However, the survival rates of KP-M1 cells did not have significant changes whether or not the bacteria were pretreated with SAL. KP organisms have been shown to remain alive after being phagocytosed [6]. Therefore, SAL treatment may only increase the uptake of the bacteria by neutrophils without affecting the extent of neutrophil-mediated intracellular killing. It is until the addition of aspirin-pretreated leukocytes that an enhance leukocyte bactericidal effect is observed.

Although the multivariate analysis showed significant protection in patients who were treated with aspirin, their blood levels of SAL were not documented. In addition, we did not perform stool sampling for KP isolation, and so the contribution of aspirin to patients’ carriage could not be assessed. It was an uncontrolled observational study. The patients who suffered from KP bacteremia may be severe and referred from other hospitals, the reliability of medication history may be a confounding factor. Therefore, we could not exclude the possibilities of other unmeasured potential confounders that may affect the association of aspirin with the enhanced leukocyte killing, thus limiting our ability to delineate the true relationship between aspirin and invasive HV-KP infection. Lastly, only one strain (KP-M1; serotype K_1_) was assessed in this study. However, epidemiological reports have indicated that capsular serotype K_1_ is the most prevalent in primary KP liver abscess [2,7]. Serotype K_1_ strains were also significantly more virulent than non-K_1_ strains, in terms of higher in vitro serum resistance and greater risk of septic ocular or central nervous system complications for primary liver abscess.^7^ Therefore, our results derived from the representative serotype K_1_ strain might be extrapolated for the majority of KP strains that caused invasive syndrome.

## Conclusions

In summary, our data indicated that SAL could affect the CPS production and the HV phenotype in KP. It also promotes the leukocyte phagocytosis and bactericidal activity against KP. The *in vitro* and *ex vivo* effects of SAL on serotype K_1_ KP warrant further investigation on the role of aspirin as an adjunct therapy for KP-mediated invasive syndrome.

## Abbreviations

CI: Confidence interval; COX: Cyclooxygenase; CPS: Capsular polysaccharide; DM: Diabetic mellitus; FITC: Fluorescein isothiocyanate; HV: Hypermucoviscosity; KP: *Klebsiella pneumoniae*; OR: Odds ratio; PG: Prostaglandin; SAL: Sodium salicylate; UA: Uronic acid.

## Competing interests

The authors declare that they have no competing interests.

## Authors’ contributions

CHL, LHS, JWL, CCC, and KDY participated in the design of this study. CHL, CCC, and RFC developed the *in vitro* assay. CHL, LHS, and KDY wrote the manuscript. All authors read and approved the final manuscript.

## Pre-publication history

The pre-publication history for this paper can be accessed here:

http://www.biomedcentral.com/1471-2334/14/47/prepub
